# A systematic review of risk of HIV transmission through biting or spitting: implications for policy

**DOI:** 10.1111/hiv.12625

**Published:** 2018-04-23

**Authors:** FV Cresswell, J Ellis, J Hartley, CA Sabin, C Orkin, DR Churchill

**Affiliations:** ^1^ Clinical Research Department London School of Hygiene and Tropical Medicine London UK; ^2^ Clinical Research Department Infectious Diseases Institute Kampala Uganda; ^3^ Lawson Unit Royal Sussex County Hospital Brighton UK; ^4^ Department of Infection and Immunity University College London London UK; ^5^ Worthing Hospital Western Sussex Hospitals NHS Foundation Trust West Sussex, Worthing UK; ^6^ Institute for Global Health University College London London UK; ^7^ Barts Health NHS Trust and Queen Mary University London The Royal London Hospital London UK

**Keywords:** bite, emergency workers, HIV, spit, transmission

## Abstract

**Objectives:**

The perceived threat of HIV transmission through spitting and biting is evidenced by the increasing use of “spit hoods” by Police Forces in the UK. In addition, a draft parliamentary bill has called for increased penalties for assaults on emergency workers, citing the risk of communicable disease transmission as one justification. We aimed to review literature relating to the risk of HIV transmission through biting or spitting.

**Methods:**

A systematic literature search was conducted using Medline, Embase and Northern Lights databases and conference websites using search terms relating to HIV, AIDS, bite, spit and saliva. Inclusion and exclusion criteria were applied to identified citations. We classified plausibility of HIV transmission as low, medium, high or confirmed based on pre‐specified criteria.

**Results:**

A total of 742 abstracts were reviewed, yielding 32 articles for full‐text review and 13 case reports/series after inclusion and exclusion criteria had been applied. There were no reported cases of HIV transmission related to spitting and nine cases identified following a bite, in which the majority occurred between family (six of nine), in fights involving serious wounds (three of nine), or to untrained first‐aiders placing fingers in the mouth of someone having a seizure (two of nine). Only four cases were classified as highly plausible or confirmed transmission. None related to emergency workers and none were in the UK.

**Conclusions:**

There is no risk of transmitting HIV through spitting, and the risk through biting is negligible. Post‐exposure prophylaxis is not indicated after a bite in all but exceptional circumstances. Policies to protect emergency workers should be developed with this evidence in mind.

## Introduction

Detailed epidemiological studies since the 1990s have provided insight into the risk of HIV transmission through sexual exposure and needlestick injuries, and have informed policy and behaviour around the use of barrier contraception, universal precautions and HIV post‐exposure prophylaxis (PEP) 1, 2, 3, 4, 5, 6, 7, 8. Recent longitudinal studies have also shown that HIV‐positive individuals on antiretroviral therapy (ART) with an undetectable plasma HIV viral load do not transmit HIV and there is increasing acceptance of the concept “undetectable = untransmissible” (U=U) 9, 10. National guidelines on HIV PEP have used these data in informing their recommendations. Provision of PEP is not recommended following potential exposure from biting and spitting; however, the risk of HIV transmission from such exposures has not been systematically evaluated 11.

In the UK, human bite injuries are a common presentation to the emergency department, comprising around 0.1% of all attendances 12. Bites represent an occupational risk to emergency workers such as policemen, paramedics, doctors and nurses, and are more likely to occur when dealing with patients with seizures, aggressive members of the public, children and those with cognitive impairment 13. In the USA there are an estimated 622 bites to emergency workers per year 14. A retrospective 4‐year review of attendees to a single UK emergency department identified 421 presentations with human bites, amounting to one every 3 days 12. Bites vary in severity from petechial haemorrhage to contusion, abrasion, laceration and avulsion 15.

Spitting represents another occupational hazard faced by emergency workers, with the Metropolitan Police alone reporting 264 spitting incidents between 2014 and 2016 16. Saliva has been shown to lyse HIV particles *in vitro* as a result of hypotonicity and many salivary proteins inhibit and inactivate HIV particles 17.

The perceived threat of HIV and other blood‐borne virus transmission through spitting and biting is evidenced by the increasing use by police forces of “spit hoods” (which are placed on potential assailants to reduce the risk of exposure to arresting officers). As of November 2016, 17 out of 49 police forces in the UK now use “spit hoods” 18. In addition, a draft parliamentary bill has called for increased penalties for assaults on emergency workers, citing the risk of communicable disease transmission as one justification 19. The draft bill also recommends mandatory provision of “intimate samples, without reasonable excuse” from those accused of spitting on emergency workers, with refusal to provide such specimens punishable as an offence. In the USA, harsh sentencing for those accused of spitting while knowingly HIV positive has been carried out, with the accused charged with causing harm by “means of a deadly weapon” 20.

We undertook a systematic literature review of HIV transmission related to biting or spitting to ensure that decisions about future policy and practice pertaining to biting and spitting incidents are informed by current medical evidence.

## Methods

### PICO (P, patient, problem or population; I, intervention; C, comparison, control or comparator; O, outcome)

The authors used the PICO framework, with the PICO “question” being formulated and answered as follows: (1) population: adults, adolescents and children; (2) intervention: bites, spitting; (3) comparator: none; (4) outcome: HIV transmission or documented absence of HIV transmission.

### Search strategy

The goal was to identify evidence relating to the risk of transmission, or lack of transmission of HIV following a biting or spitting incident. A systematic electronic search was conducted using Medline, Embase and Northern Lights databases from inception to 5 January 2018. Key natural language and controlled vocabulary search terms were used related to “HIV”, “human immunodeficiency virus”, “AIDS”, “acquired immune deficiency syndrome” AND “bites”, “bitten” OR “spit”, “spat”, “spitting”. A second search was run using the terms relating to “HIV transmission” AND “saliva”. For full search terms, see Supporting Information Notes S1. We also hand searched the British HIV Association conference abstracts from 2007 onwards and Conference for Retroviruses and Opportunistic Infections abstracts from 2014 onwards, as well as the reference lists from the papers we reviewed.

### Eligibility criteria

The following inclusion criteria were applied in article selection for full‐text review: (1) exposure of interest (biting, spitting or saliva) discussed and (2) outcome of interest described (by documented HIV antibody testing, with or without additional antigen testing, HIV viral load testing or phylogenetic analysis) or absence of HIV seroconversion (by documented negative HIV antibody test).

### Study selection

Two reviewers (JH and TR) independently conducted selection for full‐text review by applying eligibility criteria to titles and abstracts. Two reviewers (JE and FVC) then independently assessed full‐text articles for how HIV transmission had been determined and excluded articles that did not describe the exposure and outcome of interest or did not provide original case data such as narrative reviews. A list of studies for inclusion was finalized.

### Assessment of quality and data extraction

Reviewers designed a data extraction tool and independently applied it to each article. Data were extracted on study design, the perpetrator (HIV status, HIV viraemia, presence of blood in the mouth of the perpetrator, whether medically unwell and use of ART), the nature of the incident (whether biting or spitting, and the severity of the wound inflicted), the timing of HIV diagnosis, the nature of HIV testing and other HIV risk factors. Data were compared for consistency. No formal statistical analyses were undertaken in view of the nature of the studies identified.

No randomized controlled trials or cohort or case–control studies were identified, so a formal tool to assess risk of bias for the articles identified was not used. Instead, we discussed the plausibility of HIV transmission being attributable to the incident described based on documentation of baseline HIV status, the nature of the injury, the temporal relationship between the incident and a positive HIV test and phylogenetic analysis, where available. The plausibility of the incident being responsible for the subsequent HIV diagnosis was then classified as low, medium, high or confirmed based on pre‐specified criteria (Table 1). Any disagreements were resolved by consensus or a third reviewer (JH).

**Table 1:** Criteria applied to determine plausibility of HIV transmission relating to incident

	Plausibility			
	Low	Medium	High	Confirmed
Number of cases	3	2	1	3
Documented baseline negative HIV test	No	No	Yes or no	Yes or no
Temporal relationship	Positive HIV test a significant time after the incident	Positive HIV a significant time after incident	HIV seroconversion within 2 months of incident	HIV seroconversion within 2 months of incident
Phylogenetic analysis	Not done	Not done	Not done	Phylogenetic analysis suggestive of transmission
Other potential source of HIV infection	Other HIV risk factors prior to positive HIV test	No other HIV risk factors prior to positive HIV test	No other HIV risk factors	No other HIV risk factors

## Results

### Search results and study selection

**Figure 1:**
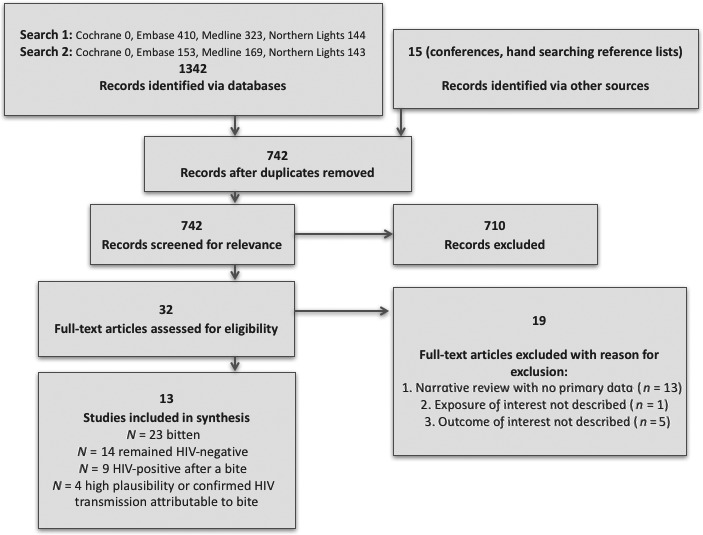
Flowchart illustrating outcomes of search citations.

Our literature search found 1357 citations: 1342 via database searches, and 15 from hand searching of conferences and reference lists. Of these, 615 were duplicates, leaving 742 for title or abstract review. A further 710 were removed because they clearly did not meet the inclusion criteria based on information contained in the title or abstract. The remaining 32 articles underwent full‐text review, of which 19 were subsequently removed because they met the exclusion criteria (no primary data, *n *=* *13; exposure of interest not described, *n *=* *1; outcome of interest not described, *n *=* *5), leaving 13 articles in the final data set (Fig. 1).

### Study characteristics and quality

Of the 13 studies selected, 11 were case reports and two were case series detailing HIV transmission, or absence of HIV transmission, following a biting episode. There were no reported cases of HIV transmission attributable to spitting. Several of the selected studies were published during the 1980s and 1990s prior to the availability of potent ART.

Of the 13 identified articles that reported alleged HIV transmission related to biting, none related to a bite in the UK and none concerned emergency care workers. The reports included information on a total of 23 people bitten by HIV‐positive individuals, of whom nine (39%) seroconverted to HIV positivity following the incident and 14 (61%) did not seroconvert (Table 2). Of these, the alleged transmissions occurred between family members (six of nine), in fights involving infliction of serious wounds (three of nine), or as a result of untrained first‐aiders placing fingers in the mouth of someone having a seizure (two of nine).

**Table 2:** Summary table of articles included in final data set

Authors [reference]	Year	Country	Exposure (nature of incident)	Outcome (HIV seroconversion)	Nature of injury	Number exposed	Blood in mouth of perpetrator	Perpetrator HIV viraemic^a^	Perpetrator on ART	Plausibility of transmission attributable to bite	Comment
Tereskerz *et al*. 14	1996	USA	Bite (HCW)	No	Skin intact	1	Unknown	Unknown	Unknown	NA	50 bites; 1.7% from a known HIV‐positive individual; no transmission reported
Tsoukas *et al*. 30	1988	Canada	Bites (HCW)	No	Skin intact (*n *=* *5), skin broken (*n *=* *3)	8	Yes	Yes	No	NA	2.5‐year follow‐up
Drummond 31	1986	Unknown	Bite (during seizure)	No	Skin broken	1	Unknown	Yes	No		18‐month follow‐up
Shirley and Ross 32	1989	USA	Bite (community, intentional)	No	Skin intact	4	Unknown	Yes	No	NA	Good follow‐up of cases
Vidmar *et al*. 21	1996	Slovenia	Bite (during seizure)	Yes	Skin broken (nail)	1	Yes	Yes	ZDV monotherapy	High	Blood in mouth from bitten tongue and a deep injury caused to nail bed. Baseline HIV test negative and seroconversion within 1 month
Akani *et al*. 28	2007	Nigeria	Bite (community, intentional)	Yes	Deep bite (lip (sutured)	1	No	Unknown	Unknown	Low	HIV test negative 1 year previously but had been sexually active in the interim. Tested HIV positive during antenatal care
Batholomew and Jones 25	2006	Trinidad	Bite (community, intentional)	Yes	Skin broken	1	Yes	Yes	No	Medium	Child tested HIV positive 4 years after being bitten by father. No baseline HIV test. No other HIV risk factors reported
CDC 22	1996	USA	Bite (community, intentional)	Yes	Multiple bites	1^b^	Yes	Unknown	No	Confirmed	HIV negative at time of bite and confirmed linkage on phylogenetics
Deshpande *et al*. 23	2011	India	Bite (community, intentional)	Yes	Deep bite (nail bed exposed)	1	No	17 163 copies/mL (plasma); 2405 copies/mL (saliva)	No	Confirmed	High‐risk injury. 91% sequence homology on phylogentic analysis
Wahn *et al*. 26	1986	Germany	Bite (community, intentional)	Yes	Skin intact	1	No	Yes	No	Medium	Child bitten by sibling 6 months prior to his death from AIDS. No baseline HIV test. Tested HIV positive after death of sibling. No other HIV risk factors reported
Anonymous 29	1987	Unknown	Bite (community, intentional)	Yes	Bite to leg	1	Yes	Yes	No	Low	HIV negative 4 years before the bite. HIV positive when tested 2 years post incident. Untraceable sexual partner. Higher risk injury as blood in mouth from teeth being knocked out
Khajotia 27	1997	Columbia	Bite (community, unintentional)	Yes	Mucosa on lip broken by kissing	1	No	Unknown	Unknown	Low	Bite is unlikely route of transmission: the biter was not confirmed to be HIV positive; recipient remained HIV negative at 7 months after bite and seroconverted 10 months after the bite
Andreo *et al*. 24	2004	Brazil	Bite (during seizure)	Yes	Deep bite (sutured)	1	Yes	Yes	No	Confirmed	Mother bitten by son during a seizure relating to AIDS‐defining illness. Seroconversion 27 days later and phylogenetic linkage

a Protagonist presumed viraemic if report is from pre‐ART era or protagonist has AIDS‐defining or critical illness in the absence of documented ART.

b Two cases described. Only one description provided original data; the other was a repetition of a case reported elsewhere.

ART, antiretroviral therapy; CDC, Centers for Disease Control and Prevention; HCW, health care worker; ZDV, zidovudine.

There was significant heterogeneity in the quality of the reports: a minority had a negative baseline HIV test in the person bitten (two of nine) or phylogenetic analysis of viruses (three of nine). Only four cases in total were classified as having high plausibility or confirmation of HIV infection being attributable to the bite.

### Highly plausible or confirmed cases of HIV transmission following bites

#### Vidmar *et al*. 21

A first aider was bitten on the hand during a seizure by a man with advanced HIV disease. The biter had confirmed blood in his mouth and was on zidovudine monotherapy, his HIV viral load (VL) was not known and he died 13 days after the incident of primary central nervous system (CNS) lymphoma. The first aider had broken skin at the site of the bite and was HIV‐negative on the day of the incident. Despite post‐exposure prophylaxis (zidovudine 1200 mg once daily), 33 days later the recipient developed an acute illness and antibody seroconversion was confirmed 54 days after the incident. The recipient had no other risk factors for HIV infection identified.

#### Centers for Disease Control and Prevention 22

A person sustained multiple bites from an HIV‐positive woman who was reported to have bleeding gums, but who had unknown HIV stage, VL and ART status. It is not reported whether the bites resulted in skin breakage. The recipient was confirmed HIV‐negative immediately after the attack and seroconverted 6 weeks later, with RNA sequencing confirming that the perpetrator and recipient shared the same viral strain.

#### Deshpande *et al*. 23

A father sustained a bite from his HIV‐positive son, causing avulsion of the thumb nail and leaving an exposed bleeding nail bed. The father was not screened for HIV at the time of the bite but presented 4 weeks later with a meningoencephalitis and was found to have acute HIV infection. The son had never received ART and had a VL of 17 163 HIV‐1 RNA copies/ml in plasma and 2405 copies/mL in saliva. There were no other risk factors for HIV transmission reported. Sequencing revealed 91% homology between perpetrator and donor HIV RNA.

#### Andreo *et al*. 24

A mother was bitten by her son in the context of a seizure. The son was subsequently diagnosed with neurotoxoplasmosis and HIV infection. Blood from a bitten tongue was present in the son's mouth at the time of the incident. The mother's wound was deep and required suturing. She was not screened for HIV at the time of the incident but presented 27 days later with fever and was found to be HIV‐positive. DNA sequencing demonstrated that viruses from the mother and son belonged to the same HIV‐1 quasi‐species.

### Medium plausibility of HIV transmission following a bite

#### Bartholomew and Jones 25

A 3‐year‐old child, born to an HIV‐negative mother, was bitten by her father who had dental caries and bleeding gums. He was found to be HIV positive 3 years later (CD4 count 4 cells/*μ*L; HIV VL not measured) and died soon afterwards. The child was therefore tested for HIV and found to be HIV positive. No other risk factors were reported. No phylogenetic analysis was undertaken.

#### Wahn *et al*. 26

A child was bitten by his brother who died 6 months after the incident and was diagnosed with toxoplasmosis and HIV infection post‐mortem (having received HIV‐infected blood during prior cardiac surgery). Family members were screened after his death and the child who had sustained the bite was found to be HIV‐positive. The bite allegedly did not result in skin breakage and there was no documentation of blood in the biting child's mouth.

### Low plausibility of HIV transmission following a bite

#### Khajotia 27

A man alleged that he contracted HIV infection from kissing during which he sustained a bite on the lip with skin breakage. He reported that the lady who bit his lip was a commercial sexual worker, although she was never confirmed to be HIV positive. He was not screened for HIV at the time of the incident but self‐reported multiple negative HIV tests in the subsequent 7 months. He was found to be HIV seropositive while undergoing investigation for gastroenteritis 10 months later. He denied any other risk factor for HIV transmission.

#### Akani *et al*. 28

During a fight, a woman was bitten on the lip by her HIV‐positive relative. The HIV stage and ART history of the perpetrator were not known, nor was it known whether she had blood in her mouth at the time of the incident. The bite resulted in a deep lip wound requiring suturing. The recipient was not tested for HIV at the time of the bite, but was found to be HIV‐positive during antenatal screening 1 year later. The recipient self‐reported a negative HIV test prior to the bite, self‐reported that her husband was HIV‐negative and denied other risk factors for HIV infection, although she had been sexually active and fallen pregnant in the interim.

#### Anonymous 29

A woman was bitten by her HIV‐positive sister during a fight. The perpetrator was known to be HIV positive and had blood in her mouth at the time of the bite, although her HIV stage, VL and ART status at the time of the incident were not reported. It was not reported whether the bite resulted in breakage of the skin. The recipient was not screened for HIV at the time of the bite, but was found to be HIV seropositive on occupational screening 2 years later. She had a documented negative HIV test 2 years prior to the bite and disclosed three sexual partners in the interim, two of whom were reportedly HIV negative but one of whom was untraceable.

## Discussion

We sought to evaluate the risk of HIV transmission from biting or spitting incidents through a systematic review of all English language literature published since the start of the HIV epidemic. Of the 742 records reviewed, there were no published cases of HIV transmission attributable to spitting, which supports the conclusion that being spat on by an HIV‐positive individual carries no possibility of transmitting HIV. Despite biting incidents being commonly reported occurrences, there were only a handful of case reports of HIV transmission secondary to a bite, suggesting that the overall risk of HIV transmission from being bitten by an HIV‐positive person is negligible. The risk of transmission of other blood‐borne viruses through biting and spitting is beyond the scope of this review and warrants further investigation.

There was significant heterogeneity in the quality of the published reports detailing HIV transmission secondary to biting episodes. Poor‐quality case reports that were published as evidence of HIV transmission secondary to a bite included those in which: (1) the recipient had no HIV‐negative test at baseline; (2) the recipient had other significant potential risk factors for HIV transmission; (3) HIV seroconversion was reported to have occurred at a time interval incompatible with transmission secondary to the bite. Therefore, of the nine reported cases of HIV infection potentially attributable to a bite, the scientific plausibility of the reports was variable and in only three cases were the attributions confirmed by RNA sequencing.

There were four cases of highly plausible HIV transmission resulting from a bite. In each case, the perpetrator had advanced HIV infection, was not on combined ART and was therefore likely to have high‐level HIV viraemia. In the majority of these cases, the bite resulted in a deep wound and the perpetrator had blood in the mouth at the time of the incident. Two cases occurred in the context of a seizure whereby an untrained first‐aid responder was bitten while trying to protect the seizing person's airway. It is therefore important that both emergency workers and first‐aid responders are trained in safe seizure management including noninvasive airway protection and use of universal precautions. It is important to note that we found no cases where an emergency care worker or police officer acquired HIV infection through being bitten.

Strengths of this systematic review include the comprehensive search strategy adopted and the clear population, intervention and outcome criteria that were adhered to. Data were extracted systematically by two independent reviewers and study quality and validity were considered and described throughout. A limitation of this review is that we only included published English language literature. More important limitations relate to the limitations of the available evidence; firstly, to date there have been no prospective studies in which the actual number of biting or spitting incidents by HIV‐positive individuals in a given time, or associated HIV seroconversions, have been documented. Secondly, two sources of bias may be important. Publication bias may potentially result in only cases of HIV seroconversion being published (significant result) as opposed to cases of no seroconversion, which could result in overestimation of the risk. Conversely, ascertainment bias, whereby individuals who have HIV‐seroconverted are not asked about biting and spitting incidents and the transmission is put down to a sexual exposure, may lead to an underestimation of the risk. The overall direction of bias is difficult to predict.

Data from England suggest that there were 89 400 people living with HIV at the end of 2016, of whom 82% had an undetectable VL, and were thus not capable of transmitting infection; this proportion has increased significantly in recent years. Current UK guidance on indications for PEP state that ‘PEP is not recommended following a human bite from an HIV positive individual unless in “extreme circumstances” and after discussion with a specialist’ 11. Necessary conditions for the transmission of HIV from a human bite appear to be the presence of untreated HIV infection, severe trauma (involving puncture of the skin), and usually the presence of blood in the mouth of the biter. In the absence of these conditions, PEP is not indicated, as there is no risk of transmission.

## Supporting information


**Notes S1.** Full search description for HIV transmission by human bite and spitting
