# Supplementary material for: A systematic review of risk of HIV transmission through biting or spitting: implications for policy
Source: HIV Med. 2018 Apr 23;19(8):532–40. doi: 10.1111/hiv.12625 (PMC6120498; doi:10.1111/hiv.12625)
Supplement: Supplementary file 1 — Notes S1. Full search description for HIV transmission by human bite and spitting [file HIV-19-532-s001.docx]

**Supplementary material**

**Full search description for HIV transmission by human bite and spitting**

Searches were carried out by an experienced clinical librarian on Ovid MEDLINE (Epub Ahead of Print, In-Process & Other Non-Indexed Citations, Daily and 1946 to Present, and Ovid Embase 1974 to 2018 Week 02. An initial search for material on HIV transmission by human bite and spitting was run on 5 January 2018, and supplemented by one on HIV transmission in saliva, executed on 9 January 2018.

Relevant natural language and controlled vocabulary terms were selected and combined, and search strategies are reproduced below. Controlled vocabulary terms were adapted where necessary from database to database. Neither MeSH nor EMTREE offer satisfactory terms for spitting, so this element was only searched for by a natural language string, and supplemented by a second broader search which used natural language and controlled vocabulary terms for saliva. Final result sets were de-duplicated and screened for relevance by the searcher, irrelevant results being discarded.

Search strategies (HIV transmission by human bite and spitting)

MEDLINE

1 "HIV INFECTIONS"/ (186469)

2 exp HIV/ (98730)

3 (hiv or hiv-1* or hiv-2* or hiv1 or hiv2 or "hiv infect*").ti,ab. (301184)

4 ("human immunodeficiency virus" or "human immunedeficiency virus" or "human immuno-deficiency virus" or "human immune-deficiency virus").ti,ab. (85397)

5 ("human immun*" and "deficiency virus").ti,ab. (611)

6 ("acquired immunodeficiency syndrome" or "acquired immunedeficiency syndrome" or "acquired immuno-deficiency syndrome" or "acquired immune-deficiency syndrome").ti,ab. (22349)

7 ("acquired immun*" and "deficiency syndrom*").ti,ab. (6148)

8 ("AIDS virus" or "HIV/AIDS").ti,ab. (28631)

9 1 or 2 or 3 or 4 or 5 or 6 or 7 or 8 (358436)

10 (spit or spitting or spat or expectorat*).ti,ab. (3970)

11 9 and 10 (172)

12 BITES, HUMAN/ (1104)

13 (bite or bites or bitten).ti,ab. (29655)

14 12 or 13 (30072)

15 9 and 14 (341)

16 "HIV INFECTIONS"/tm (26338)

17 "ACQUIRED IMMUNODEFICIENCY SYNDROME"/tm (10755)

18 DISEASE TRANSMISSION, INFECTIOUS/ (8580)

19 ((disease or virus or viral or pathogen or infection or HIV* or AIDS) adj6 (transmission or transmit*)).ti,ab. (87110)

20 16 or 17 or 18 or 19 (114091)

21 15 and 20 (194)

22 11 or 21 (365)

EMBASE

1 "HUMAN IMMUNODEFICIENCY VIRUS INFECTION"/ (243655)

2 exp "HUMAN IMMUNODEFICIENCY VIRUS"/ (171585)

3 (hiv or hiv-1* or hiv-2* or hiv1 or hiv2 or "hiv infect*").ti,ab. (340306)

4 ("human immunodeficiency virus" or "human immunedeficiency virus" or "human immuno-deficiency virus" or "human immune-deficiency virus").ti,ab. (85653)

5 ("human immun*" and "deficiency virus").ti,ab. (705)

6 ("acquired immunodeficiency syndrome" or "acquired immunedeficiency syndrome" or "acquired immuno-deficiency syndrome" or "acquired immune-deficiency syndrome").ti,ab. (21770)

7 ("acquired immun*" and "deficiency syndrom*").ti,ab. (6176)

8 ("AIDS virus" or "HIV/AIDS").ti,ab. (30633)

9 1 or 2 or 3 or 4 or 5 or 6 or 7 or 8 (437613)

10 (spit or spitting or spat or expectorat*).ti,ab. (5279)

11 9 and 10 (220)

12 BITE, HUMAN/ (4928)

13 (bite or bites or bitten).ti,ab. (32146)

14 12 or 13 (34604)

15 9 and 14 (514)

16 "VIRUS TRANSMISSION"/ (53612)

17 ((disease or virus or viral or pathogen or infection or HIV* or AIDS) adj6 (transmission or transmit*)).ti,ab. (92571)

18 16 or 17 (125778)

19 9 and 14 and 18 (180)

20 11 or 19 (396)

HIV transmission in saliva

MEDLINE:

1 *HIV INFECTIONS/tm (10617)

2 *ACQUIRED IMMUNODEFICIENCY SYNDROME/tm (4002)

3 ((disease or virus or viral or pathogen or infection or HIV* or AIDS) adj3 (transmission or transmit*)).ti,ab. (67739)

4 1 or 2 or 3 (75477)

5 *Saliva/ (21502)

6 saliva.ti. (12177)

7 5 or 6 (23776)

8 4 and 7 (227)

9 ANIMALS/ not (HUMANS/ and ANIMALS/) (4780516)

10 8 not 9 (169)

EMBASE

1 virus transmission/ and Human immunodeficiency virus infection/ (15216)

2 ((HIV* or AIDS) adj3 (transmission or transmit*)).ti,ab. (25778)

3 1 or 2 (34977)

4 saliva/ (32755)

5 saliva.ti. (11850)

6 4 or 5 (36212)

7 3 and 6 (154)

8 ANIMALS/ not (HUMANS/ and ANIMALS/) (1321042)

9 7 not 8 (153)
